# Supplementary material for: The Effects of a Glucocorticoid Receptor Agonist (GRA) on the Immune Function, Nutrient Digestibility, and Wean-to-Finish Growth Performance of Early-Weaned Pigs
Source: Animals (Basel). 2020 May 30;10(6):953. doi: 10.3390/ani10060953 (PMC7341203; doi:10.3390/ani10060953)
Supplement: Supplementary file 1 [file animals-10-00953-s001.pdf]

animals

MDPI

Table S1: Effects of glucocorticoid receptor agonist (GRA), in-feed antibiotic (ANT) sex, and time (day) on complete blood cell count (CBS), concentration of plasma proteins, and measures of blood chemistry during the first week post-weaning of pigs.

	GRA-				GRA+				SE	*p* ≤
	ANT-		ANT+		ANT-		ANT+			*GRA×ANT×SEX×DAY*
	Gilt	Barrow	Gilt	Barrow	Gilt	Barrow	Gilt	Barrow		
Total WBC (K/µl)										
Day 1	15.4	10.6	13.1	13.8	11.8	10.4	11.8	13.1	2.40	0.09
Day 2	12.3	14.3	13.5	15.5	13.4	10.5	9.9	15.2	2.35	-
Day 4	15.8	16.5	11.4	16.7	13.5	13.4	12.8	15.6	2.88	-
Day 7	30.4	24.7	22.5	33.4	15.5	22.0	22.2	23.4	4.55	-
Neutrophils (K/µl)										
Day 1	5.1	3.8	7.8	6.4	5.3	6.9	3.4	5.0	5.27	0.82
Day 2	8.4	8.9	9.1	9.1	9.1	6.4	25.6	10.2	8.40	-
Day 4	9.1	7.9	6.3	10.3	8.3	8.2	6.7	7.1	9.11	-
Day 7	15.2	14.4	14.7	24.6	8.2	13.5	12.5	15.0	15.20	-
Lymphocytes (K/µl)										
Day 1	7.7	7.3	5.9	6.5	8.1	5.7	6.6	7.5	1.27	0.77
Day 2	6.1	3.9	4.0	3.8	3.5	2.8	3.4	2.9	1.29	-
Day 4	5.5	4.7	4.1	5.4	3.6	3.2	5.4	7.1	1.58	-
Day 7	6.2	9.7	5.0	7.4	6.8	7.8	7.7	6.1	2.24	-
N: L ratio										
Day 1	0.7	0.5	1.4	1.0	1.0	1.3	0.5	0.5	0.28	0.95
Day 2	1.3	2.2	2.5	2.4	2.8	2.8	7.1	4.0	1.67	-
Day 4	1.9	1.6	1.7	2.0	2.4	2.1	1.4	1.2	0.67	-
Day 7	2.5	1.5	3.1	3.3	1.4	1.9	1.2	2.4	1.15	-
Monocytes (K/µl)										
Day 1	0.4	0.3	0.4	0.3	0.4	0.4	0.2	0.2	0.17	0.17
Day 2	0.3	0.2	0.6	0.9	0.5	0.5	0.6	0.6	0.32	-
Day 4	0.5	0.5	0.8	0.8	0.9	0.8	0.6	0.8	0.29	-
Day 7	1.6	0.5	2.0	1.7	0.5	0.7	1.4	1.9	0.57	-
Eosinophils (K/µl)										
Day 1	0.7	0.5	0.5	0.5	0.2	0.4	0.4	0.4	0.24	0.07
Day 2	0.4	0.3	0.4	0.4	0.3	0.3	0.2	0.5	0.17	-
Day 4	0.3	0.3	0.1	0.4	0.4	0.4	0.4	0.6	0.17	-
Day 7	2.8	1.8	0.8	0.5	0.5	1.2	1.0	0.5	0.28	-
RBC (M/µl)										
Day 1	6.9	6.3	7.1	7.0	7.1	7.1	6.9	7.1	0.40	0.60
Day 2	7.3	7.2	6.8	6.7	6.9	6.9	6.5	7.3	0.35	-
Day 4	6.3	6.7	7.1	6.9	7.0	7.1	6.6	7.2	0.35	-
Day 7	6.6	6.8	7.5	7.4	7.9	6.8	7.2	6.2	0.56	-

Table S1. Continued.

	GRA-				GRA+				SE	$p \leq$
	ANT-		ANT+		ANT-		ANT+			*GRA×ANT×SEX×DAY*
	Gilt	Barrow	Gilt	Barrow	Gilt	Barrow	Gilt	Barrow		
HGB (g/dL)										
Day 1	11.4	9.7	11.2	11.4	10.8	12.3	9.7	10.8	1.32	0.40
Day 2	11.2	11.8	10.2	9.6	9.9	10.2	9.1	10.8	1.05	-
Day 4	10.0	9.7	10.4	10.3	10.5	10.0	9.3	10.2	0.97	-
Day 7	10.9	9.8	11.0	11.9	11.8	10.0	9.7	9.2	1.50	-
HCT (%)										
Day 1	39.6	34.6	40.3	40.3	38.3	45.2	35.5	38.3	5.06	0.44
Day 2	41.2	43.7	35.5	36.4	35.9	39.5	32.1	40.5	4.18	-
Day 4	35.3	35.4	38.9	36.2	37.9	36.6	35.1	37.4	3.90	-
Day 7	38.6	36.1	40.3	43.6	42.7	34.6	36.1	33.2	6.17	-
Platelet (%)										
Day 1	692	798	768	813	952	668	789	982	145.6	0.81
Day 2	591	869	788	814	793	747	775	647	140.1	-
Day 4	619	501	775	682	876	1311	624	1004	134.5	-
Day 7	919	552	740	725	1106	816	717	917	187.0	-
Plasma protein (g/dl)										
Day 1	6.4	6.4	5.5	6.2	5.3	4.5	6.5	5.8	0.48	0.34
Day 2	6.2	5.1	5.4	5.9	5.7	4.8	6.2	5.8	0.37	-
Day 4	5.6	6.1	5.4	5.3	5.4	4.8	6.0	5.7	0.34	-
Day 7	6.0	5.8	5.4	5.8	5.1	5.1	5.7	5.2	0.68	-
Plasma fibrinogen (mg/dl)										
Day 1	600^{y}	520^{y}	517	530^{yz}	444^{y}	500^{y}	547	528^{y}	76.4	0.02
Day 2	700^{xy}	569^{y}	615	447^{z}	475^{y}	484^{y}	461	696^{x}	78.1	-
Day 4	715^{x}	741^{x}	525	592^{xy}	623^{x}	593^{y}	646	714^{x}	67.2	-
Day 7	787^{x}	759^{x}	656	719^{x}	660^{x}	739^{x}	654	361^{z}	76.2	-
Electrolyte balance (mEq/L)										
Day 1	37.0	35.7	34.8	35.6	37.1	36.8	35.4	37.7	1.81	0.57
Day 3	40.3	41.0	42.9	42.4	39.5	39.0	38.3	38.5	1.46	-
Day 5	38.1	41.4	42.3	40.3	41.9	40.0	40.8	39.9	1.45	-
Blood glucose (mg/dL)										
Day 1	118	165	114.0	131	114	118	107	132	14.2	0.18
Day 3	106	114	121.5	111	141	122	109	149	12.2	-
Day 5	104.0	112	106.4	108	108	106	105	102	15.1	-
Blood urea nitrogen (mg/dl)										
Day 1	9.5	8.0	9.0	10.3	4.5	5.6	6.0	5.5	2.41	0.76
Day 3	8.8	9.0	7.3	9.0	9.8	8.0	7.8	5.4	2.11	-
Day 5	9.5	12.0	7.6	5.6	6.4	5.9	9.5	5.6	2.16	-

Table S1. Continued.

	GRA-				GRA+				SE	*p* ≤
	ANT-		ANT+		ANT-		ANT+			*GRA×ANT×SEX×DAY*
	Gilt	Barrow	Gilt	Barrow	Gilt	Barrow	Gilt	Barrow		
Creatinine (mg/dl)										
Day 1	1.4	1.3	1.5	1.3	1.0	1.1	1.1	1.1	0.11	0.35
Day 3	1.2	1.2	1.1	1.2	1.2	1.2	1.2	1.2	0.10	-
Day 5	1.4	1.1	1.2	1.2	1.0	1.3	1.1	1.2	0.10	-
Anion gap (mmol/L)										
Day 1	16.3	16.5	15.6	17.7	16.6	16.8	16.5	18.7	1.26	0.45
Day 3	16.3	17.8	17.3	17.4	17.6	16.4	15.3	17.4	1.09	-
Day 5	16.8	18.2	18.0	18.0	16.8	17.6	17.3	16.0	1.09	-

[1]Data are least squares mean (± largest SE) and represent the best estimate of mean based on repeated measurement analysis of variance; ND = not detectable

[a-c]Means within a row lacking a common superscript are different ($p < 0.05$).

[x-z]Means within a column lacking a common superscript letter are different ($p < 0.05$).

GRA+ pigs received 0.2 mg/kg BW dexamethasone -1 and 3 days post-weaning; ANT+ pigs received 110 ppm tylosin in the diet for the first week post weaning

RBC= red blood cell; WBC = white blood cell.

Table S2: Effects of glucocorticoid receptor agonist (GRA), in-feed antibiotic (ANT), sex, and time (day) on intestinal mucosal morphology, the relative concentrations of selected jejunum enzymes and protein, and the digestibility of dietary energy and protein in early-weaned pigs.

	GRA-				GRA+				SE	*p* ≤
	ANT-		ANT+		ANT-		ANT+			*GRA×ANT×SEX×DAY*
	Gilt	Barrow	Gilt	Barrow	Gilt	Barrow	Gilt	Barrow		
Villus height (mm)										
Day 1	0.28	0.25	0.27	0.25	0.24	0.23	0.27	0.24	0.021	0.32
Day 3	0.26	0.27	0.29	0.30	0.24	0.24	0.25	0.23	0.020	-
Day 5	0.23	0.28	0.28	0.31	0.26	0.21	0.26	0.24	0.025	-
Crypt depth (mm)										
Day 1	0.20	0.21	0.19	0.23	0.23	0.21	0.19	0.19	0.023	0.72
Day 3	0.24	0.25	0.26	0.23	0.24	0.25	0.23	0.24	0.023	-
Day 5	0.24	0.26	0.29	0.29	0.33	0.30	0.32	0.27	0.023	-
Villus density (villi/mm)										
Day 1	9.9	8.8	10.1	9.2	10.1	10.8	9.6	10.2	0.55	0.66
Day 3	8.5	8.7	9.7	9.3	10.7	9.3	8.7	8.3	0.60	-
Day 5	8.9	8.6	8.6	8.8	8.6	8.8	9.1	9.7	0.60	-
Villus height-to-crypt depth ratio										
Day 1	1.45	1.24	1.47	1.22	1.09	1.18	1.48	1.30	0.135	0.62
Day 3	1.10	1.08	1.14	1.34	1.04	0.97	1.09	1.00	0.135	-
Day 5	1.01	1.09	0.98	1.09	0.90	0.76	0.83	0.92	0.135	-
Surcease (pg/mg protein)										
Day 1	86	54	41	32	67	86	46	48	8.9	0.27
Day 3	129	139	147	146	131	139	156	141	9.6	-
Day 5	137	127	124	135	118	112	120	119	9.0	-
Maltase (ng/mg protein)										
Day 1	5.8	4.6	6.0	5.7	8.3^{xy}	8.5^{xy}	8.6^{x}	7.8^{x}	1.61	0.04
Day 3	6.5	7.6	8.4	8.3	5.9^{y}	6.4^{y}	3.5^{y}	3.0^{y}	1.88	-
Day 5	7.1	7.1	6.7	7.0	10.1^{x}	9.6^{x}	10.4^{x}	10.2^{x}	2.25	-
Aminopeptidase N (pg/mg protein)										
Day 1	89^{y}	65^{y}	191^{y}	$108x^{y}$	79.3^{y}	94^{y}	98^{y}	165^{x}	26.5	0.01
Day 3	54.2^{y}	71^{y}	91^{z}	91^{y}	123.0^{x}	86^{y}	131^{xy}	98^{y}	17.1	-
Day 5	171.5^{x}	164^{x}	239^{x}	249^{x}	117^{xy}	171^{x}	181^{x}	131^{xy}	29.8	-
Alkaline phosphatase (ng/mg protein)										
Day 1	18.5	20.6	16.9	13.9	15.3	20.5	10.8	13.9	2.76	0.13
Day 3	16.2	17.7	15.1	11.1	21.8	18.8	20.7	21.1	2.89	-
Day 5	16.3	25.0	8.5	11.3	14.9	20.7	ND	ND	3.04	-

Table S2. Continued.

	GRA-				GRA+				SE	$p \leq$
	ANT-		ANT+		ANT-		ANT+			*GRA×ANT×SEX×DAY*
	Gilt	Barrow	Gilt	Barrow	Gilt	Barrow	Gilt	Barrow		
Heat shock protein 70 (ng/mg protein)										
Day 1	ND	ND	ND	ND	ND	ND	ND	ND	-	0.01
Day 3	ND	ND	ND	ND	ND	ND	ND	ND	-	-
Day 5	149.8[c]	150.9[c]	160.6[b]	ND	273.8[a]	274.6[a]	ND	ND	41.66	-
ATTD of gross energy										
Day 7	0.47	0.40	0.44	0.44	0.56	0.50	0.47	0.52	0.045	0.80
Day 14	0.57	0.53	0.54	0.55	0.61	0.59	0.67	0.63	0.059	-
Day 21	0.54	0.60	0.59	0.63	0.68	0.69	0.61	0.63	0.060	-
Day 28	0.69	0.69	0.72	0.74	0.73	0.76	0.76	0.73	0.056	-
AID of crude protein										
Day 1	0.70[c]	0.72[c]	0.80[ab]	0.99[a]	0.94[a]	0.90[a]	0.74[bc]	0.27[d, y]	0.114	0.02
Day 3	0.78[ab]	0.76[ab]	0.77[ab]	0.85[ab]	0.88[ab]	0.92[a]	0.71[b]	0.75[b, x]	0.141	-
Day 5	0.75[c]	0.83[abc]	0.96[a]	0.84[abc]	0.96[ab]	0.86[abc]	0.84[abc]	0.78[bc, x]	0.082	-

[1]Data are least squares mean (± largest SE) and represent the best estimate of mean based on repeated measurement analysis of variance; ND = not detectable.

[a-c]Means within a row lacking a common superscript letter are different ($p < 0.05$).

[x-z]Means within a column lacking a common superscript letter are different ($p < 0.05$).

GRA+ pigs received 0.2 mg/kg BW dexamethasone -1 and 3 days post-weaning; ANT+ pigs received 110 ppm tylosin in the diet for the first week post weaning.

ATTD = apparent total tract digestibility; AID = apparent ileal digestibility.

© 2020 by the author. Licensee MDPI, Basel, Switzerland. This article is an open access article distributed under the terms and conditions of the Creative Commons Attribution (CC BY) license (http://creativecommons.org/licenses/by/4.0/).
